# Hemodialysis-induced changes in hematocrit, hemoglobin and total protein: Implications for relative blood volume monitoring

**DOI:** 10.1371/journal.pone.0220764

**Published:** 2019-08-12

**Authors:** Leszek Pstras, Malgorzata Debowska, Alicja Wojcik-Zaluska, Wojciech Zaluska, Jacek Waniewski

**Affiliations:** 1 Nalecz Institute of Biocybernetics and Biomedical Engineering, Polish Academy of Sciences, Warsaw, Poland; 2 Department of Physical Therapy and Rehabilitation, Medical University of Lublin, Lublin, Poland; 3 Department of Nephrology, Medical University of Lublin, Lublin, Poland; University of Nottingham School of Medicine, UNITED KINGDOM

## Abstract

**Background:**

Relative blood volume (RBV) changes during hemodialysis (HD) are typically estimated based on online measurements of hematocrit, hemoglobin or total blood protein. The aim of this study was to assess changes in the above parameters during HD in order to compare the potential differences in the RBV changes estimated by individual methods.

**Methods:**

25 anuric maintenance HD patients were monitored during a 1-week conventional HD treatment. Blood samples were collected from the arterial dialysis blood line at the beginning and at the end of each HD session. The analysis of blood samples was performed using the hematology analyzer Advia 2120 and clinical chemistry analyzer Advia 1800 (Siemens Healthcare).

**Results:**

During the analyzed 30 HD sessions with ultrafiltration in the range 0.7–4.0 L (2.5 ± 0.8 L) hematocrit (HCT) increased by 9.1 ± 7.0% (mean ± SD), hemoglobin (HGB) increased by 10.6 ± 6.3%, total plasma protein (TPP) increased by 15.6 ± 9.5%, total blood protein (TBP) increased by 10.4 ± 5.8%, red blood cell count (RBC) increased by 10.8 ± 7.1%, while mean corpuscular red cell volume (MCV) decreased by 1.5 ± 1.1% (all changes statistically significant, p < 0.001). HGB increased on average by 1.5% more than HCT (p < 0.001). The difference between HGB and TBP increase was insignificant (p = 0.16).

**Conclusions:**

Tracking HGB or TBP can be treated as equivalent for the purpose of estimating RBV changes during HD. Due to the reduction of MCV, the HCT-based estimate of RBV changes may underestimate the actual blood volume changes.

## Introduction

Relative blood volume (RBV) changes during hemodialysis (HD) are typically estimated from continuous (or quasi-continuous) non-invasive measurements of optical, electrical, acoustic or viscous properties of blood flowing through the dialyzer lines [1, 2] The three most common methods of estimating RBV changes during HD are based on measurements of: 1) hematocrit, 2) hemoglobin concentration in the whole blood, or 3) total blood protein concentration [2, 3] (see Table 1). Based on such measurements the RBV changes can be calculated from the following equations:

$$\Delta BV_{HCT}(t)=\left(\frac{HCT_{0}}{HCT_{t}}-1\right)\times 100\;[\%]$$ (1)

$$\Delta BV_{HGB}(t)=\left(\frac{HGB_{0}}{HGB_{t}}-1\right)\times 100\;[\%]$$ (2)

$$\Delta BV_{TBP}(t)=\left(\frac{TBP_{0}}{TBP_{t}}-1\right)\times 100\;[\%]$$ (3)

where at any time point t, the value of hematocrit (HCT), whole blood hemoglobin (HGB) or total blood protein concentration (TBP) are related to the respective quantity measured at initial time point 0.

**Table 1:** Examples of commercially available systems for monitoring relative blood volume changes during hemodialysis.

System	Manufacturer	Measured variable	Sensor type	Operation principle
Crit-Line IV	Fresenius Medical Care AG & Co, Bad Homburg, Germany	hematocrit, O_2_ saturation	optical	multiple wavelengths of light absorbed and scattered by different blood constituents
Blood Volume Monitor (BVM)	Fresenius Medical Care AG & Co, Bad Homburg, Germany	total blood protein	acoustical	ultrasonic pulses transmitted across the blood sample
Hemoscan	Gambro AB, Stockholm, Sweden	whole blood hemoglobin	optical	monochromatic light absorbed/transmitted across the blood sample

Similarly, based on changes in total plasma protein concentration (TPP), the relative changes in apparent plasma volume (PV) can be calculated as follows:

$$\Delta PV_{TPP}(t)=\left(\frac{TPP_{0}}{TPP_{t}}-1\right)\times 100\;[\%]$$ (4)

All above methods/equations assume a good mixing of blood across the whole cardiovascular system and the extracorporeal circuit, so that the parameter changes measured at the dialyzer blood line can be treated as representative of their changes at the whole-body level. All methods assume also a constancy of the total amount of the measured quantity in the circulatory system (e.g. a constant amount of HGB or TPP) and obviously neither of them can detect sudden changes in blood volume (BV) without hemoconcentration (e.g. due to hemorrhage or blood leakage in the extracorporeal circuit).

The HCT-based method carries some additional assumptions. Firstly, the method assumes that the total volume of red blood cells (RBCs) in the circulatory system (including the extracorporeal circuit) remains constant throughout dialysis, which requires not only a constant number of RBCs available in the circulation (i.e. no sequestration or release of RBCs in the spleen and a negligible or balanced erythropoiesis and erythrocyte lysis), but also the lack of osmotic water shifts between plasma and RBCs (note that there are other possible cases satisfying the assumption of a constant total volume of RBCs, such as a lower number of larger erythrocytes or a higher number of smaller erythrocytes). Secondly, it assumes that the ratio of whole-body HCT to central HCT (known as the F-cells ratio [4]) also remains constant, so that any changes in HCT measured at the dialyzer blood line are consistent with the global HCT changes on the whole-body level. The assumptions related to pooling or release of blood with different level of HCT at different sites of circulation or to sequestration of erythrocytes in the spleen is important also for estimating RBV changes from changes in HGB or TBP, given that the amount of erythrocytes circulating in the system affects the amount of HGB in blood.

Due to the above assumptions, the accuracy of monitoring RBV changes to describe absolute BV changes is still under debate [3,4,2,5,6]. The aim of this paper was to analyze changes of HCT, HGB and TBP in patients undergoing routine maintenance HD in order to assess how the estimations of RBV changes may differ depending on the monitoring method.

## Materials and methods

### Patients and dialysis settings

The presented data come from end-stage renal disease anuric patients with arteriovenous fistula undergoing maintenance, thrice-weekly HD with duration of approximately 4 hours. The data were collected during 3 consecutive HD sessions of a 1-week dialysis treatment with the interdialytic breaks before the sessions of 3, 2, and 2 days respectively. The original dataset included data from 25 patients (75 HD sessions in total). For the purpose of this study, we selected from this dataset only the sessions during which the patients did not receive any fluid infusions, nor did they consume any drinks or food (to avoid any confounding influence on BV changes), the dialyzer settings were not changed throughout the session and the dataset did not have any missing values. We identified a total of 30 of such ‘undisturbed’ HD sessions in 12 patients (age 63 ± 12 years, range: 44–79, 8 females). For 7 patients all three sessions were included in the analysis, whereas for the other 5 patients only one or two sessions were analyzed. The priming saline present in the extracorporeal circuit before each dialysis was in all cases infused to the patient when the circuit was filled with the patient’s blood. The composition of the dialysis fluid, the dialyzer blood flow rate and the ultrafiltration rate (all varying between the patients and the analyzed sessions–see Table 2) were kept constant throughout each session. In all cases the dialysate flow rate was set to 500 mL/min and its temperature was kept at 36°C. The dialysis treatment was delivered using mainly low-flux dialyzers, except for two patients (four HD sessions) in which high-flux dialyzers were used. During all dialysis sessions the patients remained in the supine position. The whole group of patients has already been subject of the studies on phosphate, urea, and creatinine clearances [7], phosphate kinetics [8,9], extracellular calcium mass balance [10] and transcapillary transport of fluid and proteins during HD [11,12].

**Table 2:** Patient characteristics and dialysis settings for the first (HD 1), second (HD 2), third (HD 3) and all HD sessions. All data presented as mean ± SD (range).

	Symbol	HD 1	HD 2	HD 3	ALL
Number of patients (females/males)	N	11 (8/3)	9 (6/3)	10 (7/3)	12 (8/4)
Age, years	-	62.6 ± 12.4 (44–79)	62.9 ± 10.8 (46–79)	66.2 ± 10.3 (46–79)	63.1 ± 11.9 (44–79)
Time on dialysis, years	-	10.3 ± 9.0 (1–25)	9.9 ± 7.8 (1–23)	6.9 ± 6.3 (1–17)	9.8 ± 8.8 (1–25)
Number of sessions analyzed	n	11	9	10	30
Dialysis duration, min	T	237 ± 13 (210–255)	237 ± 15 (210–255)	236 ± 12 (210–255)	237 ± 13 (210–255)
Dialyzer ultrafiltration, mL	UF	3000 ± 639 (1700–4000)	2333 ± 762 (1000–3800)	2070 ± 874 (700–3600)	2435 ± 880 (700–4000)
Blood flow rate, mL/min	Q_b_	274 ± 50 (200–350)	269 ± 54 (200–350)	263 ± 46 (200–350)	270 ± 48 (200–350)
Dialysate sodium, mmol/L ^†^	Na_d_	142 ± 2 (140–144)	143 ± 2 (140–146)	143 ± 3 (139–149)	142 ± 2 (139–149)
Dialysate potassium, mmol/L ^†^	K_d_	2.5 ± 0.7 (1.1–3.2)	2.5 ± 0.7 (1.1–3.4)	2.5 ± 0.7 (1.1–3.2)	2.5 ± 0.7 (1.1–3.4)
Dialysate calcium, mg/dL ^†^	Ca_d_	5.4 ± 0.5 (4.7–6.1)	5.6 ± 0.6 (4.9–6.5)	5.4 ± 0.4 (4.8–6.1)	5.5 ± 0.5 (4.7–6.5)
Dialysate magnesium, mg/dL ^†^	Mg_d_	1.3 ± 0.1 (1.2–1.6)	1.3 ± 0.1 (1.2–1.4)	1.4 ± 0.2 (1.2–2.0)	1.3 ± 0.2 (1.2–2.0)

^†^ measured in dialysate inflow.

### Ethics statement

The study was approved by the Bioethical Committee at the Medical University of Lublin (Poland) and written informed consent was obtained from each patient.

### Blood tests

The arterial blood samples were collected from the arterial HD line at the beginning and at the end of each dialysis session.

The hematological analysis of blood samples was performed using the automatic hematology analyzer Advia 2120 (Siemens Healthcare, Erlangen, Germany) based on the measurement methods described in [13,14], as follows. The red blood cell count (RBC) and mean corpuscular volume of red blood cells (MCV) are measured utilizing the principles of flow cytometry with hydrodynamic focusing and laser light scattering, preceded by isovolumetric cell sphering with a special reagent containing sodium dodecyl sulphate and glutaraldehyde, thus eliminating the cell shape variability factor [13] (MCV is obtained as mean value of the red blood cell volume histogram). The hemoglobin concentration (HGB) in the blood (following erythrocyte lysis) is measured using the cyanide-free hemoglobin method with colorimetrical optical readings [13,15]. According to the technical specification of the Advia 2120 system, the coefficient of variation for the measurements of RBC, MCV and HGB are 1.2%, 0.78% and 0.93% respectively. The hematocrit is calculated by Advia 2120 as the product of RBC and MCV in the analyzed sample. The measurements of TPP and plasma sodium concentration were performed using the clinical chemistry analyzer Advia 1800 (Siemens Healthcare, Erlangen, Germany).

TBP was calculated as the sum of HGB and TPP corrected for HCT. All data were combined for the first (HD 1), second (HD 2) and third (HD 3) dialysis of the week.

### Statistical analysis

Data are presented as mean ± standard deviation (SD) and statistical significance was set at the level of p-value < 0.05, unless otherwise indicated. The variables were compared by the Student’s t-test. Statistical dependence between variables was tested using the Spearman's correlation coefficient (R).

## Results

**Fig 1:**
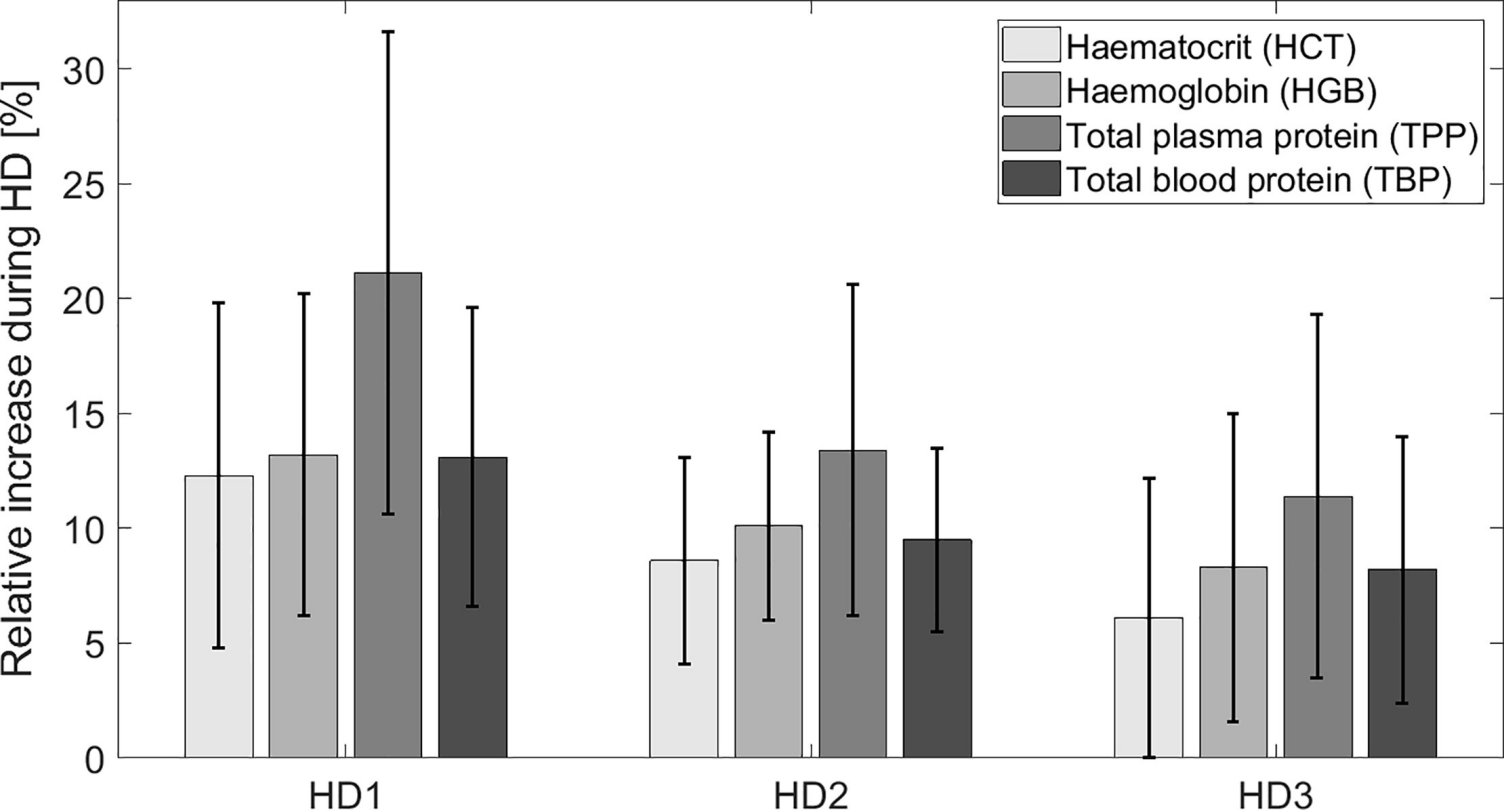
Dialysis-induced changes in hematocrit, hemoglobin, total plasma protein and total blood protein. Relative increase (mean ± SD) of hematocrit (HCT), hemoglobin (HGB), total plasma protein (TPP) and total blood protein including hemoglobin (TBP) during three consecutive HD sessions in the week in the analyzed group of patients based on the laboratory measurements of arterial blood samples taken at the beginning and at the end of each session (see Table 3). For all three averaged HD sessions the differences in dialysis-induced relative increase between HCT and HGB, and between HGB and TPP were all statistically significant (p < 0.02), except for the difference between HCT and HGB in the first HD session (p = 0.23). The changes of HGB and TBP during HD 1 and HD 3 were almost equal (p = 0.81 and p = 0.63, respectively); for HD 2 there was a significant (p < 0.02), but very small difference (0.6%).

The values of HCT, HGB, TPP, TBP, MCV and RBC as well as the plasma sodium concentration at the beginning and at the end of dialysis averaged for HD 1, HD 2 and HD 3 are presented in Table 3. The relative increase of HCT, HGB, TPP and TBP during dialysis is shown in Fig 1.

**Table 3:** Blood parameters measured in the studied patients at the beginning (subscript 0) and at the completion (subscript end) of first (HD 1), second (HD 2), third (HD 3) and all HD sessions. All data presented as mean ± SD (range).

	Symbol	HD 1	HD 2	HD 3	ALL
Hematocrit, %	HCT_0_	35.2 ± 4.0 (29.8–43.8)	36.0 ± 4.3 (30.1–44.2)	34.7 ± 3.2 (29.0–39.8)	35.3 ± 3.8 (29.0–44.2)
	HCT_end_	39.4 ± 4.2*** (34.1–48.1)	39.1 ± 5.3*** (32.7–47.3)	36.8 ± 4.2* (32.0–45.6)	38.4 ± 4.6*** (32.0–48.1)
Hemoglobin concentration in the blood, g/dL	HGB_0_	11.4 ± 1.3 (9.9–14.4)	11.6 ± 1.4 (9.9–14.5)	11.2 ± 1.0 (9.6–13.2)	11.4 ± 1.2 (9.6–14.5)
	HGB_end_	12.9 ± 1.4*** (11.0–16.0)	12.7 ± 1.8*** (11.0–15.7)	12.1 ± 1.4** (10.8–15.4)	12.6 ± 1.5*** (10.8–16.0)
Total plasma protein concentration, g/dL	TPP_0_	6.4 ± 0.6 (4.8–7.2)	6.5 ± 0.7 (5.1–7.4)	6.6 ± 0.6 (5.4–7.5)	6.5 ± 0.6 (4.8–7.5)
	TPP_end_	7.8 ± 0.8*** (6.7–8.9)	7.5 ± 0.9*** (6.0–8.6)	7.4 ± 0.7** (6.8–8.7)	7.5 ± 0.8*** (6.0–8.9)
Total blood protein concentration, g/dL	TBP_0_	15.6 ± 1.3 (13.3–18.3)	15.7 ± 1.5 (13.5–18.6)	15.5 ± 1.1 (13.4–17.3)	15.6 ± 1.2 (13.3–18.6)
	TBP_end_	17.6 ± 1.4*** (15.8–20.3)	17.2 ± 1.8*** (15.0–19.9)	16.7 ± 1.5** (15.7–19.8)	17.2 ± 1.5*** (15.0–20.3)
Mean corpuscular volume of red blood cells, fL	MCV_0_	94.5 ± 4.3 (87.0–100.6)	94.3 ± 3.8 (90.4–100.7)	94.9 ± 3.3 (90.8–99.7)	94.6 ± 3.7 (87.0–100.7)
	MCV_end_	93.1 ± 4.0** (87.2–99.1)	93.1 ± 3.3** (89.3–99.0)	93.3 ± 3.5*** (88.6–98.5)	93.2 ± 3.5*** (87.2–99.1)
Red blood cell count, x 10^6^/μL	RBC_0_	3.7 ± 0.4 (3.3–4.7)	3.8 ± 0.4 (3.3–4.7)	3.7 ± 0.4 (3.2–4.4)	3.7 ± 0.4 (3.2–4.7)
	RBC_end_	4.2 ± 0.5*** (3.6–5.2)	4.2 ± 0.6*** (3.5–5.1)	3.9 ± 0.5* (3.4–5.2)	4.1 ± 0.5*** (3.4–5.2)
Plasma sodium concentration, mmol/L	Na_pl,0_	142 ± 3 (138–147)	141 ± 3 (138–146)	140 ± 2 (137–143)	141 ± 3 (137–147)
	Na_pl,end_	141 ± 2 (137–143)	141 ± 2 (138–142)	141 ± 2 (137–144)	141 ± 2 (137–144)

***

** and

* denote p-value < 0.001, < 0.01 and < 0.05, respectively, vs. the beginning of HD.

Looking at the combined data from all 30 analyzed HD sessions, HCT increased during dialysis on average by 9.1 ± 7.0% (mean ± SD), HGB increased by 10.6 ± 6.3%, TPP increased by 15.6 ± 9.5%, and TBP increased by 10.4±5.8% (all changes were statistically significant, p < 0.001). Thus, during dialysis HGB increased on average by 1.5 ± 0.3% (mean ± standard error) more than HCT (p < 0.001), whereas TBP increased on average by 1.3 ± 0.4% (mean ± standard error) more than HCT (p < 0.01). The difference between HGB change and TBP change for all sessions combined was very small (0.3%) and insignificant (p = 0.16). As expected, pairwise correlations between dialysis-induced changes in HCT, HGB, TPP and TBP were all very strong (r > 0.9, p < 0.001). The changes of HCT during all analyzed HD sessions featured a higher variability assessed by the coefficient of variation (CV = 77%) compared to the changes of HGB (CV = 59%), TPP (CV = 61%) or TBP (CV = 56%).

**Fig 2:**
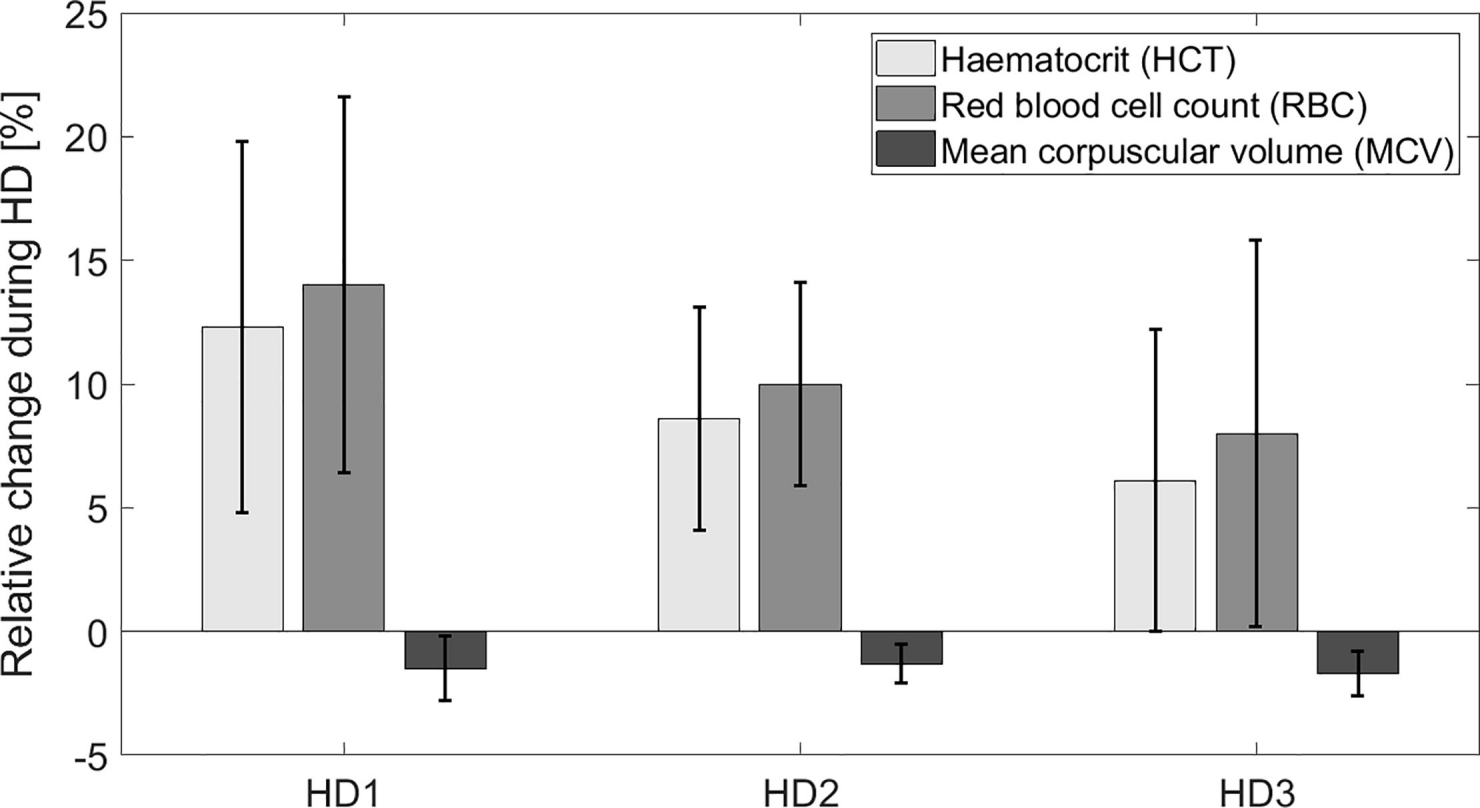
Dialysis-induced changes in red blood cell parameters. Relative change (mean ± SD) of hematocrit (HCT), red blood cell count (RBC) and mean corpuscular volume of red blood cells (MCV) during three consecutive hemodialysis sessions in the week in the analyzed group of patients based on the laboratory measurements of arterial blood samples taken at the beginning and at the end of each session (see Table 3).

Fig 2 shows the relative changes of HCT, RBC and MCV during dialysis. In all HD sessions combined RBC increased by 10.8 ± 7.1% (p < 0.001), whereas MCV decreased by 1.5 ± 1.1% (p < 0.001). Relative changes of RBC and MCV were statistically significant in all three HD sessions (p < 0.01). The difference in relative change between HCT and RBC was also statistically significant for all HD sessions (p < 0.01). The changes of RBC and HGB were almost equal (p = 0.65), which suggests that the discrepancy between relative changes of HGB and HCT is due to the reduction in MCV.

**Fig 3:**
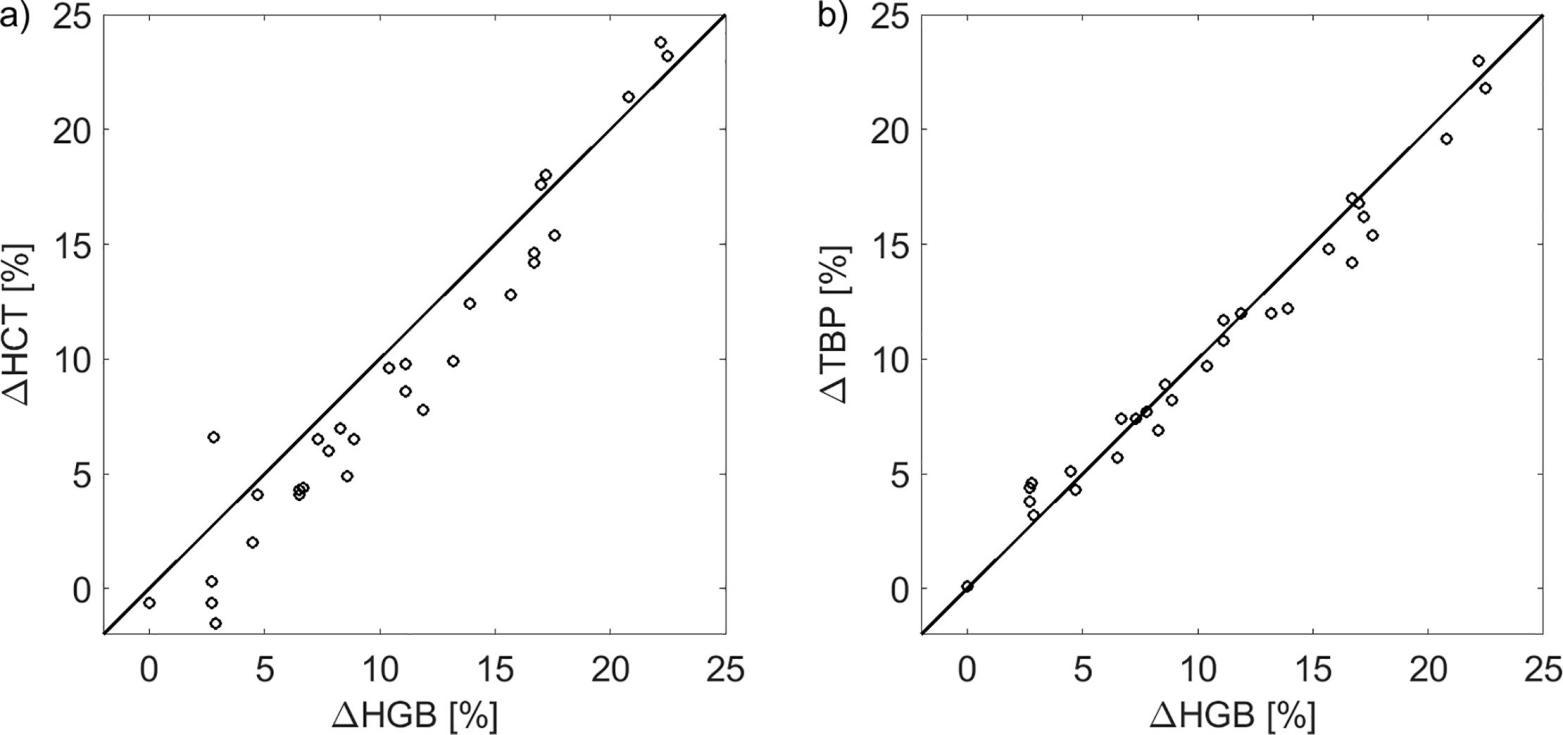
Correlation between dialysis-induced changes in hematocrit, hemoglobin and total blood protein. Relative changes of hemoglobin (ΔHGB) paired with a) relative changes of hematocrit (ΔHCT) and b) relative changes of total blood protein (ΔTBP) during all analyzed hemodialysis sessions plotted against the identity line.

Fig 3A shows the relative changes of HCT versus the relative changes of HGB during all analyzed dialysis sessions. For the vast majority of cases ΔHCT is lower than ΔHGB with the data points lying below the identity line. The paired relative changes of TBP and HGB lie, in general, much closer to the identity line, as shown in Fig 3B. Interestingly, almost all cases with ΔHCT higher than ΔHGB lie on the right side of Fig 3A, which could suggest that for larger relative changes of HCT and HGB the effect of the relatively lower increase of HCT compared to HGB disappears or is even slightly reversed. Such a bias, however, was not confirmed by the Bland-Altman analysis of the relationship between the difference (ΔHGB-ΔHCT) and the magnitude of changes (average of ΔHGB and ΔHCT), the correlation of which was not significant (p = 0.06).

**Fig 4:**
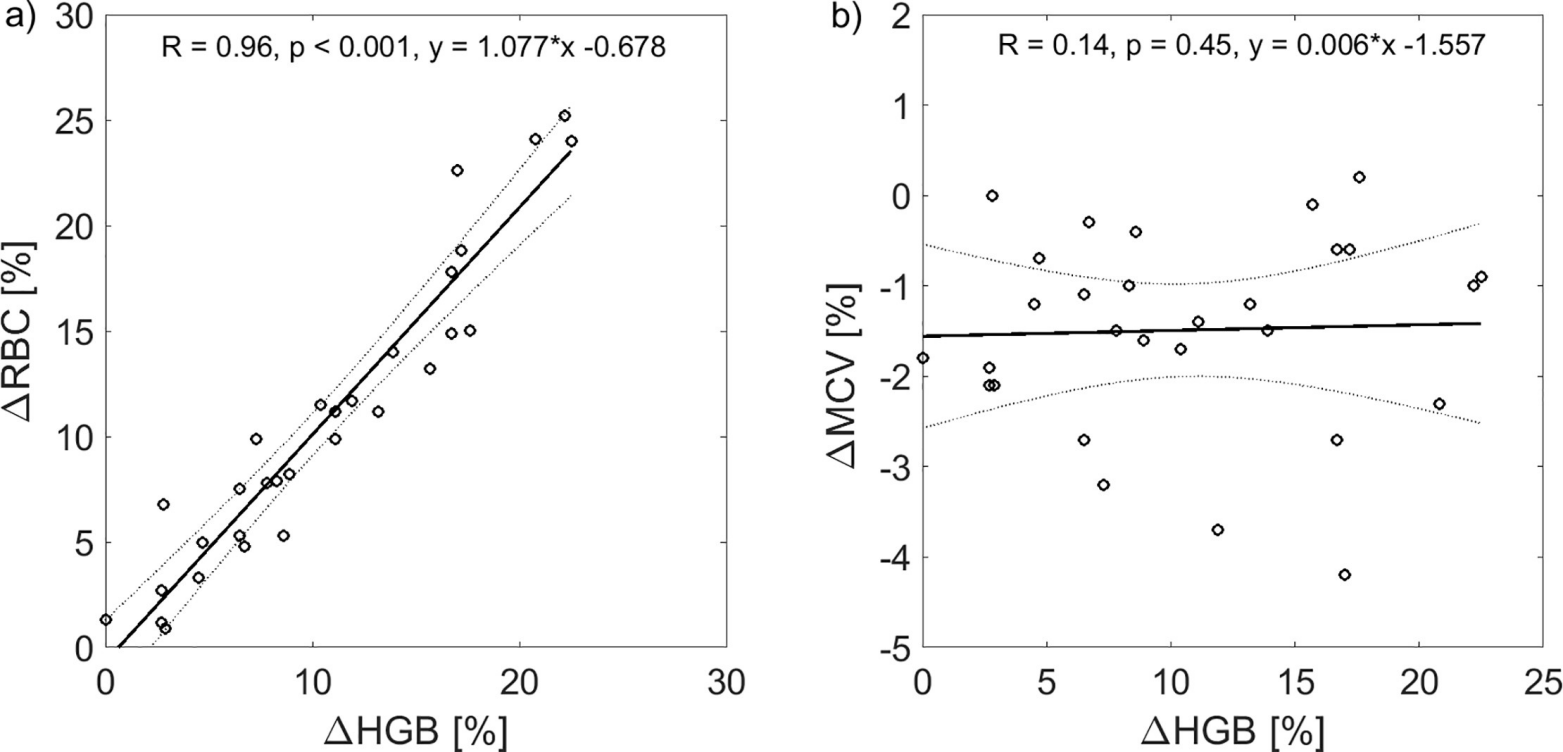
Correlation between dialysis-induced changes in red blood cell count, hemoglobin and mean corpuscular red cell volume. Relative changes of hemoglobin (ΔHGB) paired with a) relative changes of red blood cell count (ΔRBC) and b) relative changes of mean corpuscular red cell volume (ΔMCV) during all analyzed hemodialysis sessions with linear regression fit (solid lines) and 95% confidence bounds (dotted lines).

Relative changes of RBC and HGB were strongly correlated (R = 0.96, p < 0.001) and lying relatively close to the identity line (see Fig 4A), whereas relative changes of MCV and HGB were uncorrelated (p = 0.45, see Fig 4B), which further shows that there was no magnitude-related bias in the discrepancy between ΔHGB and ΔHCT.

**Fig 5:**
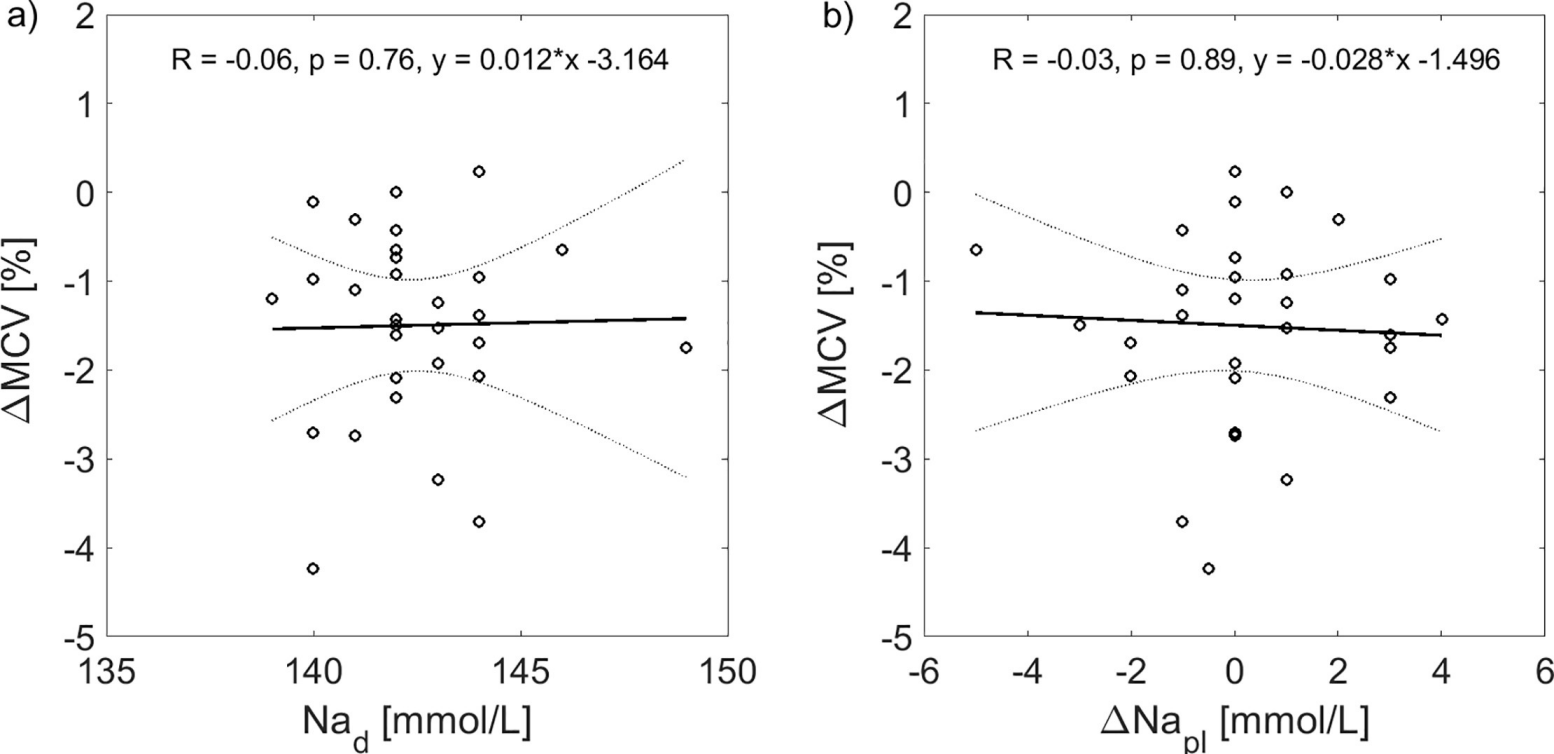
Correlation between dialysis-induced changes in mean corpuscular red cell volume, dialysate sodium concentration and change in plasma sodium concentration during dialysis. Dialysis-induced changes in mean corpuscular red cell volume (ΔMCV) paired with a) dialysate sodium concentration (Na_d_) and b) change in plasma sodium concentration (ΔNa_pl_) during all analyzed hemodialysis sessions with linear regression fit (solid lines) and 95% confidence bounds (dotted lines).

The dialysis-induced changes in MCV were not correlated with the dialysate sodium concentration (see Fig 5A), nor with the change in plasma sodium concentration during dialysis (see Fig 5B).

**Fig 6:**
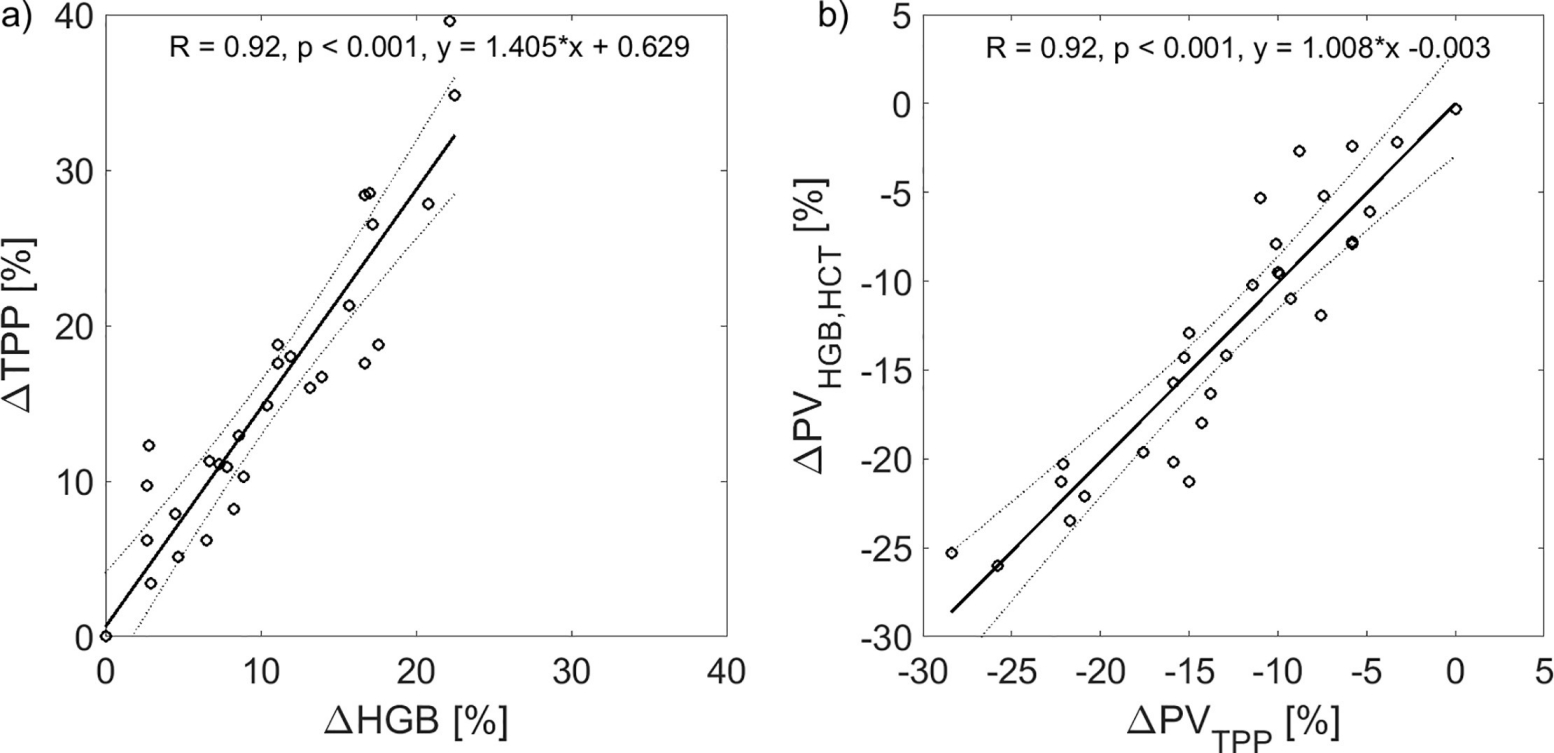
Correlation between dialysis-induced changes in total plasma protein, hemoglobin and plasma volume estimates. a) Relative changes of hemoglobin (ΔHGB) paired with relative changes of total plasma protein (ΔTPP); b) Comparison between the relative plasma volume (PV) changes calculated from changes in total protein concentration (TPP, see Eq 4) or calculated from changes in whole blood hemoglobin (HGB) and hematocrit (HCT), assuming the constant F-cells ratio of 0.9. Data shown for all hemodialysis sessions with linear regression fit (solid lines) and 95% confidence bounds (dotted lines).

As shown in Fig 6A, the correlation between the relative changes in TPP and HGB was high (R = 0.92, p < 0.001). The correlation between the relative changes in PV calculated from changes in TPP (Eq 4) or calculated from changes in HCT and BV (the latter based on HGB changes, (Eq 2), assuming the constant F-cells ratio of 0.9 [16,17], was also high (R = 0.92, p < 0.001, see Fig 6B). The average difference between the relative changes in PV calculated using the two above methods was very small (0.1%) and insignificant (p = 0.86).

## Discussion

The main finding of this study is that HGB increased during HD relatively more than HCT. The difference between the HGB increase and HCT increase (1.5 percentage point on average) can be explained by the concomitant decrease in MCV of a similar percentage magnitude (1.4 fL on average, see Table 3) leading to the reduced total red cell volume (assuming the constant number of erythrocytes in the circulation).

Given that HCT was calculated by Advia 2120 as the product of RBC and MCV, the relative change of HCT during HD can be expressed as:

$$\Delta HCT=\frac{HCT_{end}-HCT_{0}}{HCT_{0}}=\Delta RBC+\Delta MCV+\Delta RBC\cdot \Delta MCV$$ (5)

where:

$$\Delta RBC=\frac{RBC_{end}-RBC_{0}}{RBC_{0}}$$ (6)

$$\Delta MCV=\frac{MCV_{end}-MCV_{0}}{MCV_{0}}$$ (7)

If MCV remained unchanged during HD (as typically assumed), the last two terms of Eq (5) would become 0 and the relative HCT changes would follow changes in RBC, thus being dependent purely on the hemoconcentration effect of HD. In such a case, the relative changes of HCT should follow those of HGB. If, however, MCV does change during dialysis, in theory the sum of the last two terms of Eq (5) should reflect the difference in the observed relative changes of HCT and HGB. In this study, for all analyzed HD sessions combined, the difference between the sum of the last two terms of Eq (5) (ΔMCV + ΔRBC ∙ ΔMCV) and the disparity between the relative increase of HCT and HGB (ΔHCT - ΔHGB) was, indeed, very small (0.14 percentage points) and insignificant (p = 0.72). Our study suggests hence that tracking HCT or HGB changes is not entirely equivalent for the purpose of assessing the RBV changes during HD, and that the RBV changes calculated from HCT variation in the arterial blood line may underestimate the absolute BV reduction due to osmotic water shifts from RBCs to plasma.

It should be noted that the average dialysis-induced reduction in MCV or the difference between HCT and HGB increase reported in this study, while statistically significant, are relatively small (1.5%), and hence not that significant from the clinical point of view. These effects, however, may be of more significance in some individual patients (e.g. the reduction of MCV by circa 4% was observed in two patients in this study, see Fig 4B).

Similar level of MCV decrease during HD was already reported by a few authors in the past [18,19,20]. In the study by Fleming et al. [21], a higher MCV decrease (by 3.8%) was reported for HD with a high dialysate sodium concentration (154 mmol/L), while an increase of MCV by 2.5% was found at a very low dialysate sodium concentration (126 mmol/L), indicating that the dialysis fluid composition, by affecting plasma sodium level and plasma osmolarity, significantly influences changes in MCV during dialysis. In the present study we did not observe correlation between the dialysis-induced changes in MCV and the dialysate sodium concentration (see Fig 5A) possibly due to the relatively narrow range of dialysate sodium concentration (4 mmol/L range for 90% of cases). Note also that during a typical dialysis MCV may transiently increase while the erythrocytes pass through the dialyzer [22].

Even though our study showed no significant difference between changes in HGB and TBP, the TBP-based method of estimating RBV changes may be additionally biased by a possible variation of the amount of plasma proteins within the circulatory system due to protein refilling from the interstitium during HD (through the lymphatic system and transcapillary fluid absorption) [23,24,25,26]. Apparently this was not the case in the present study, given that the relative PV changes estimated from TPP variation were highly correlated with the PV changes calculated from HGB and HCT variation with the regression line being very close to the identity line (see Fig 6B).

It should be mentioned that our study has certain limitations. Firstly, as indicated in Methods, HCT was calculated by the automatic analyzer Advia 2120 as the product of RBC and MCV in the analyzed blood sample. This means that the aforementioned disparity between HCT and HGB increase during HD is not independent from the dialysis-induced change in MCV, to which it was compared. However, measuring HCT by centrifuging blood (micro hematocrit method) could entail measurement error due to trapped plasma (especially in case of abnormalities in RBCs [27]), whereas measuring HCT using blood gas analyzers could entail errors due to abnormal levels of electrolytes and TPP often seen in dialysis patients [28].

Secondly, during measurements of MCV by Advia 2120, blood is diluted with a special reagent, which may cause some osmotic water shifts from/to erythrocytes in case of a difference in osmolarity between the blood sample and the reagent. Before dialysis patients have typically an increased blood osmolarity (due to uremia), and hence in contact with the fluid with a lower osmolarity, erythrocytes could be subject to swelling before they are isovolumetrically sphered and “lightly fixed” with glutaraldehyde present in the reagent. This effect can potentially contribute to the observed difference in MCV before and after dialysis. However, given that the difference in the effective osmolarity between the blood sample and the reagent should not be very high, the potential effect of erythrocyte swelling should be relatively small and possibly partly compensated by the concomitant solute exchange between erythrocytes and the “new” plasma diluted by the reagent. Note that the above phenomenon is not limited to the analyzer used in this study, but is present in all automatic cell count analyzers, in which the blood sample is diluted. Moreover, the automatic hematological analysis can be erroneous in case of abnormalities in the red blood cell size distribution histogram, due to alterations in erythropoiesis or iron deficiency seen in dialysis patients [29].

As already mentioned, all three methods of estimating RBV changes discussed in this paper depend on the amount of erythrocytes available in the circulation and their distribution among different parts of the circulatory system described by the F-cells ratio. Although some authors reported an increase of the F-cells ratio during HD [30,31], more recently it has been shown that it remains relatively stable during normal HD conditions [4], and so it is believed that it should not affect the estimation of RBV changes derived from centrally measured hematological variables, such as central HCT [4]. As far as the amount of erythrocytes available in the circulation is concerned, it has been shown that during HD, erythrocytes are released to the circulation from the splanchnic and splenic bed [32], which constitutes an additional confounding factor in estimating BV changes from HCT, HGB or TBP. On the other hand, the assumption of a constant volume of RBCs affects only the RBV change estimation based on HCT changes.

Regardless of the method used to monitor RBV changes, one should remember that there are several other factors affecting BV changes during dialysis, such as changes in body position, food intake, hydration status, intra-dialytic exercise or administration of intravenous fluids [2,3,4], and that the RBV changes may feature intra-individual variability not linked to differences in ultrafiltration volume [33]. Also, the characteristics of blood (such as oxygen saturation) and dialysis settings (such as blood flow rate or dialyzer inflow pressure) may affect the accuracy of RBV monitors [34]. One should also remember that the values provided by all RBV monitors refer to BV changes relative to the start of the measurements, at which point the patient’s body may not be in the steady-state conditions in terms of fluid and blood distribution [2].

Moreover, every RBV monitor can be characterized by its own accuracy of measuring a given parameter and thus the accuracy of estimating RBV changes during HD. Dasselaar et al. compared three popular RBV monitors listed in Table 1, i.e. Crit-Line, Hemoscan and Blood Volume Monitor and found significant differences both between the RBV changes estimated by individual devices, as well as between the RBV estimated by these devices and the RBV calculated from laboratory measurements of HGB [34]. Moreover, Dasselaar et al. showed that the difference between individual devices depend on the magnitude of BV changes [34]. Given the above and the aforementioned intrinsic limitations of estimating BV changes from measurements of HCT, HGB or TBP, the indications of RBV monitors should be always treated only as approximate estimates of the actual BV changes. The latter can be measured by a standard dilution technique with radioisotope tracers (e.g. radio iodinated albumin and ^51^Cr-labeled red blood cells) [17], but as a more feasible alternative, absolute BV changes during HD may be estimated based on RBV recordings before and after a step change in ultrafiltration rate [35,36,37,38,39] or following dialysate infusion [40,41,42].

In conclusion, due to possible changes of MCV during dialysis, tracking HCT, HGB or TBP variations is not entirely equivalent for the purpose of assessing RBV changes during HD. Assuming the relatively stable ratio between the whole-body hematocrit and central hematocrit, variation of the latter can still be used as a basis for estimating BV changes during HD, but such an estimation should be ideally corrected for the changes in red cell volume, as previously suggested by Fleming et al. [21], either by some empirical formula or through online measurements of MCV.
